# Deficits in response inhibition on varied levels of demand load in anorexia nervosa: an event-related potentials study

**DOI:** 10.1007/s40519-018-0558-2

**Published:** 2018-08-28

**Authors:** Ling Yue, Yingying Tang, Qing Kang, Qian Wang, Jijun Wang, Jue Chen

**Affiliations:** 1 Department of Clinical Psychology, Shanghai Mental Health Center, Shanghai Jiao Tong University School of Medicine, 600 Wan Ping Nan Road, Shanghai, 200030 People’s Republic of China; 2 Department of EEG and Imaging, Shanghai Mental Health Center, Shanghai Jiao Tong University School of Medicine, 600 Wan Ping Nan Road, Shanghai, 200030 People’s Republic of China; 3 Med-X Research Institute, School of Biomedical Engineering, Shanghai Jiao Tong University, Shanghai, 200030 People’s Republic of China

**Keywords:** Event-related potentials, Stop-signal task, N2, P300, Anorexia nervosa, Response inhibition.

## Abstract

**Purpose:**

The aim of the present study was to investigate the executive function of inhibitory control in anorexia nervosa (AN), which is considered as an underlying pathophysiology of restricting eating.

**Methods:**

In this work, we examined the function of response inhibition in 27 unmedicated AN patients and 30 healthy controls (HC) using stop-signal tasks with different demand loads. Two event-related potentials (ERP) during the stop-signal tasks, N2 and P300, were compared between the AN and HC groups.

**Results:**

We found attenuated P300 amplitudes and delayed N2 latencies in AN patients across all three demand loads compared to HCs. We also found significant interaction between group and level of demand load. N2 latencies were prolonged when the inhibitory demand was lower in the AN group, whereas no differences in N2 latencies were found across different demand loads in HCs.

**Conclusions:**

Taken together, altered P300 amplitudes and N2 latencies may be associated with impaired response inhibition in AN patients. In particular, alterations of fronto-central N2 activations were demand-related, which might contribute to an aberrant inhibitory control process in AN.

**Level of evidence:**

Level II, controlled trial without randomization.

**Electronic supplementary material:**

The online version of this article (10.1007/s40519-018-0558-2) contains supplementary material, which is available to authorized users.

## Introduction

Anorexia nervosa (AN) is an eating disorder characterized by restrained eating, being significantly underweight, disturbance in self-perception of weight/shape, and intense fear of gaining weight or becoming fat (American Psychiatric Association, DSM-V, 2013) [1, 2]. The disease commonly begins during adolescence in young females, its lifetime prevalence is 3.6% [3] and it has a high mortality rate of 20% [4]. Furthermore, AN in young men is also common; the lifetime prevalence is 0.24%, higher than expected, which has not been sufficiently noted [5]. People with AN who have distorted views of their body shapes tend to over-control food intake and relentlessly pursue thinness [6]. Though the aetiology of AN remains unknown [1, 6, 7], deficits in executive function (i.e., inhibitory control in particular) are considered to play a key role in the pathophysiology of the disease [8–13]. Neuropsychological studies have reported lower rates of motor impulsivity [9] and greater subjective self-control in AN patients compared with healthy controls [10]. In terms of behavioural studies, empirical research has demonstrated that impairments in set-shifting were consistently observed in patients with AN [14, 15], indicating increased cognitive control manifested as inflexible patterns of thinking. Another behavioural study using a delayed discounting task found excessive reward-related inhibitory control in acutely ill AN patients [11]. However, these findings have not always been consistent, for example, studies also reported no differ in the behavioural measures of impulsiveness between AN and healthy control [6, 12, 16]. In addition, another behavioural study reported a deficient motor inhibition in patients with AN [13]. Moreover, behavioural impulsivity and subjective self-control coexisting were also reported in AN [10]. Nonetheless, these findings support the suggestion that AN patients show impaired inhibition control that is more complex than what is captured by a single behavioural measure alone.

Evidence from neuroimaging studies suggest that the impaired inhibitory control in AN is associated with alterations in two distinct neural pathways [1, 2, 17–19]. Reduced activations have been found within the ventral neuro-circuit including the striatum, the amygdala, the hippocampus, and the cerebellum [8]. These regions, which are associated with somatic states, affective relevance and interoceptive awareness, are hypoactive in AN patients, resulting in deficient appetite [20, 21]. The dorsal loop, which includes the anterior cingulate cortex (ACC), the dorsal prefrontal cortex (DLPFC), the medial prefrontal cortex (mPFC), and the orbitofrontal cortex, is also altered in individuals with AN, thus affecting their attention, working memory, arousal and emotional processing, as well as reward processing [17, 22, 23]. However, fMRI findings in AN patients during a behavioural response shifting task were inconsistent. One study showed a hyperactive fronto-parietal network indicating effortful and supervisory cognitive control in AN patients [24], while other studies showed reduced prefrontal activity [2, 24, 25]. Interestingly, a fMRI study using different inhibitory control task demands found that the activity of the mPFC was demand-specific in recovered AN individuals [6]. When the inhibitory control demands became more difficult, AN individuals showed less mPFC activation. In summary, inhibition control plays a critical role in AN, and the mechanisms underlying the neurobiology remain largely unclear, especially in currently ill AN patients.

The stop-signal paradigm is a well-designed and commonly used task for examining the capacity for response cancellation, which is one form of response inhibition [26, 27]. The horse-race model is used to interpret the Go/Stop task. The Go signal evokes the process of stimulus identification and the preparation for a response. The following Stop signal includes the response inhibition [27]. Compared to the Go/No-Go task, the stop-signal task is a more direct measure of response cancellation that is specifically designed to evaluate motor inhibition during its execution. The Go/No-Go task measures another form of inhibition called response restraint, which the ability to withhold before a response has been started and which has a decision-making component [28]. Hence, the stop-signal task may be a purer method for testing the response inhibition process. ERP studies provide more evidence about the neural mechanisms of response cancellation [26, 29]. There are two typical ERP components (i.e., N2 and P300) associated with the Stop signal [27]. The N2 is linked to the inhibition of the motor plan prior to motor execution. The component is more pronounced on the stop-signal trials than on no-go-signal trials [26, 30]. The frontal N2 is followed by the central P300 which is associated with monitoring of the outcome of the inhibition process [30]. It is known that amplitude modulations of a component reflect variability in the “degree” of process activation, while latency reflects the temporal properties of process activation [31]. Thus, poor inhibitory control may be reflected in lower amplitude and prolonged latency, leading to weaker processing and taking longer to cancel a response. A recent study explored neural activity of the food-related Go/No-Go task among individuals with different BMI status (normal, over weight and obesity) [32]. When inhibiting responses to high-calorie food compared to low-calorie food, larger N2 and P300 amplitudes were found, suggesting that the N2/P300 were sensitive to the extent of inhibitory control independent of BMI status. To the best of our knowledge, there is only one electrophysiological study on response inhibition in AN individuals. The study used the 3-Stimulus Oddball Task to investigate response inhibition and found no alteration component when comparing AN patients with healthy controls [33]. However, the paradigm has been mostly linked with attention shifting to changes in the environment, partly representing inhibition control [34]. The N2/P300 complex has been found to be altered and related to anxiety-related personality traits and obsessive–compulsive disorder [30, 35]. Since the personality research in AN has revealed an anxious and obsessive–compulsive temperament in patients, this approach might be applicable for exploring electrophysiological evidence for deficits in response inhibition in AN patients, and thus helping researchers to understand the etiology of the disease [20, 25].

Despite the evidence recovered AN patients showed aberrant inhibitory control processes, no direct brain electrical physiological study has explored this mechanism in currently ill AN patients. In the present study, we use the event-related potential (ERP) to precisely track temporal changes during the stop-signal task with different levels of inhibitory demand in currently ill AN patients. We hypothesized that (1) lower fronto-central N2/P300 amplitudes and longer latencies evoked by Stop signal would be observed in AN patients, which reflects deficient response inhibition compared to controls, and (2) between-group differences of N2/P300 activations may depend on demand loads, and such impaired inhibitory processing may be more server on the hard condition. We also hypothesized that ERP activations would be sensitive to the clinical characteristics of the AN group, which would provide more evidence about the neural mechanism of AN.

## Methods

### Subjects

Twenty-seven unmedicated AN patients were recruited from the Outpatient Department of Shanghai Mental Health Center (SHMC) for this study. All patients were required to meet the following criteria: (1) DSM-IV diagnosis of AN, (2) 14 years or older, (3) female, and (4) 6 years of education or more. The exclusion criteria were (1) the diagnosis of severe physical illness which might affect cognitive functions, (2) the diagnosis of other mental disorder besides AN, (3) the diagnosis of neurological disease, (4) a history of current medication treatment or substance abuse, (5) a family history of a DSM-IV Axis I mental disorder, and (6) high-risk suicide thoughts or behaviours. The clinical assessments and ERP experiments were completed immediately after patients agreed to join the study. After that, regular therapeutic services were given, such as DBT treatment or antidepressant according to each patient’s situation.

Thirty gender-, age- and education-matched healthy controls (HCs) were recruited via advertisements on the social media tool WeChat. The general exclusion criteria were the same as the AN group. Additional inclusion criteria for HCs were (1) without lifetime diagnosis of DSM-IV Axis I mental disorder, (2) no history of psychiatric medication or substance abuse, (3) having a score of < 20 on the Eating Attitudes Test (EAT-26), which indicates a low risk of developing an eating disorder, (4) having a score of < 19 on the Beck Depression Inventory (BDI), and (5) having a score of < 45 on the Beck Anxiety Inventory (BAI).

All subjects were Han Chinese females, were right-handed and had normal or corrected-to-normal vision. Detailed demographic and clinical characteristics are summarized in Table 1. Informed consent was obtained from all individual participants included in the study. The present study was approved by the Ethics Committee of the Shanghai Mental Health Centre. All participants (or their parents if they were under 18 years of age) provided written informed consent. The study was carried out in accordance with the Declaration of Helsinki.

**Table 1:** Demographic and clinical characteristics of both groups (*M*, SD) separated by individuals with AN and healthy controls

	AN (*n* = 27)	HC (*n* = 30)	*t, P*
Age (years)	19.00 (4.58)	19.23 (3.59)	− 0.22
School education (years)	12.60 (2.81)	12.9 0 (3.04)	− 0.40
BMI	14.52 (2.03)	20.45 (2.46)	− 9.89**
EAT-26	18.00 (16.45)	4.43 (4.42)	6.25**
BDI	18.00 (16.45)	4.47 (4.38)	4.15**

**< 0.001, significant group difference (two-tailed *t* test)

### Stimulation and procedure

**Fig. 1:**
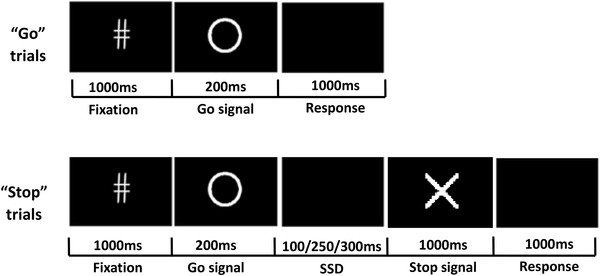
The procedure for both the “Go” and “Stop” trials

A stop-signal task was constructed according to the typical experiment [4] and administered to each subject in a pseudo-random order. There were 4 blocks of 100 trials in each task, and 50% of the trials were “Go” and the other 50% were “Stop”. The procedures for the Go and Stop trials are both shown in Fig. 1. In the Go trials, a fixation was presented for 1000 ms and followed by a circle of 200 ms. The subjects were asked to press the button labelled “1” as soon as they saw the circle. The response window lasted 1000 ms. In the Stop trials, the 200 ms circle was followed by a stop-signal delay (SSD). Then, the stop stimuli cross showed up and lasted for 200 ms. The subjects were asked not to respond to the circle followed by the cross. The SSD appeared for 100 ms, 250 ms or 300 ms in a pseudo-random order corresponding to the proportions of 30%, 40% and 30%, respectively. The procedure was programmed using Eprime 2.0. Each subject responded via a response box, and their behavioural measures were recorded on a computer [Intel (R) Core(TM) 2 Duo CPU E7500 @2.93 GHz with 2.00 GB memory].

### Recordings and analysis

The electroencephalogram (EEG) data were recorded from 63 electrodes mounted in an elastic cap (Easycap, Brain Products Inc., Bavaria, Germany) with the tip of the nose as the reference. The electrooculogram (EOG) data were recorded using two electrodes above the right eye and below the left eye. The EEG signals were recorded using Vision Recorder (BrainAmp, Brain Products Inc., Bavaria, Germany). The impedance for each electrode was kept below 5 kΩ and the sampling rate was 1000 Hz.

EEG data were further analysed off-line using Brain Analyzer (Version 1.05, Brain Products Inc., Bavaria, Germany). Artifacts from vertical and horizontal eye movements and blinks were removed by an ocular correction algorithm [36]. The artifact-free data were processed using a zero phase-shift IIR band-pass filter (24db/Oct) between 0.05 and 30 Hz. Data were then segmented into 1400 ms epochs starting 200 ms before the onset of the Stop signals. Segments were corrected using the − 200 to 0 ms pre-stimulation baseline. Segments with amplitudes over ± 100 µV were rejected.

Artifact-free segments were averaged across each SSD (100 ms, 250 ms and 300 ms) for the Stop trials. The N2 and the P300 components were measured as the most negative peaks within the 180–250 ms time window and the most positive peak within the 250–320 ms time window, respectively, from post-stimulation at the medial frontal electrodes (Fz, FCz and Cz). Individual N2 and P300 peak amplitudes and latencies were obtained for each SSD in each participant from the average waveforms.

### Statistical analysis

Behavioural accuracies for the Stop trials were statistically analysed with a repeated-measures analysis of variance (ANOVA) for the within-group factor “SSD” (i.e., 100 ms, 250 ms and 300 ms) and the between-group factor “Group” (i.e., AN and HC). Accuracy and reaction time for the Go trials were tested with independent *t*-tests for group differences.

A repeated-measures ANOVA was applied to evaluate the group effect on the N2/P300 amplitudes and latencies, respectively. The between-group factor was “Group”, and the within-group factors were “SSD” and “Location” (i.e., Fz, FCz and Cz). When there was a significant main effect of SSD or Location, post hoc t tests with Bonferroni correction were performed.

The Pearson correlation between the N2/P300 amplitudes and the clinical characteristics, including BMI, scores on the BDI, EAT-26, and HAMD, was computed. The same correlation was also computed between the N2/P300 latencies and clinical characteristics. The analyses were conducted using SPSS v19.0, and the significance level was set at *P* < 0.05.

## Results

### Performance measurement

Behavioural accuracy and reaction time were measured in 25 AN subjects and 30 HCs. Behavioural records of two AN subjects were missing.

In the Stop trials, there was no significant Group difference in accuracy [*F*(2,53) = 0.04, *P* = 0.84]. However, the effect of SSD was significant [*F*(1,53) = 87.42, *P* < 0.001]. Post hoc *t* tests with Bonferroni correction showed that the accuracy for SSDs of 100 ms was the highest (94.9%), followed by the 250 ms (83.1%) and the 300 ms (74.3%). The Group × SSD interaction was not significant [*F*(2,52) = 1.293, *P* = 0.382].

In the Go trials, there was a significant difference between the AN and the HC groups on accuracy [*t*(53) = − 3.11, *P* < 0.01]. The accuracy in AN subjects (89.5%) was lower than that in HC (96.4%) individuals. There was no significant Group difference in reaction time for the Go trials [*t*(53) = − 1.65, *P* = 0.11].

### ERP waveforms

**Fig. 2:**
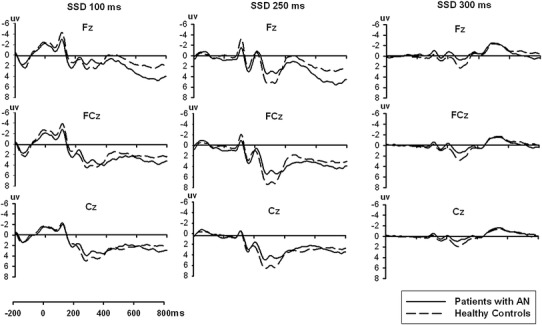
Grand average waveforms at electrodes Fz, FCz and Cz for each SSD in patients with anorexia nervosa and healthy controls

Grand average waveforms for each SSD at the Fz, FCz and Cz electrodes in both groups are depicted in Fig. 2. The ERP waveform components were analysed and are reported in Table 2.

**Table 2:** N2/P300 components on the stop-signal task between groups with different SSD and Location

	P300 amplitude	P300 latency	N2 amplitude	N2 latency
Group	**5.69 (0.02)**	0.83 (0.37)	0.65 (0.42)	**5.50 (0.02)**
SSD	**75.45 (0.00)**	2.94 (0.06)	**3.56 (0.04)**	0.71 (0.49)
Location	**13.10 (0.00)**	**12.87 (0.00)**	**44.05 (0.00)**	**13.47 (0.00)**
Group × SSD	0.762 (0.47)	1.73 (0.18)	0.45 (0.63)	**3.33 (0.04)**
Group × Location	0.086 (0.92)	0.26 (0.72)	0.30 (0.67)	0.12 (0.84)
SSD × Location	**15.22 (0.00)**	0.24 (0.89)	**17.13 (0.00)**	0.96 (0.42)
Group × SSD × Location	0.194 (0.94)	1.74 (0.15)	0.76 (0.51)	0.46 (0.73)

Values are given as *F* value (*p* value)

Values in bold are statistically significant at an alpha of 0.05

Repeated measures analysis of variance indicated that the Group effect on the P300 amplitude was statistically significant [*F*(1,55) = 5.69, *P* < 0.05]. The P300 amplitudes elicited by patients with AN (3.34 ± 0.42 µV) were significantly lower than those elicited by HC (4.73 ± 0.40 µV). The effect of SSD was also significant [*F*(2,110) = 75.45, *P* < 0.01]. The SSD of 250 ms (6.15 ± 0.45 µV) elicited a much larger P300 amplitude than the SSD of 100 ms (4.00 ± 0.36 µV, *P* < 0.001) and the SSD of 300 ms (1.95 ± 0.20 µV, *P* < 0.001). Location also had a significant effect [*F*(2,110) = 13.10, *P* < 0.01] with a maximum found at FCz (4.65 ± 0.34 µV). The interaction of SSD and Location was also significant [*F*(4,220) = 15.22, *P* < 0.001]. There was no significant interaction of Group × SSD, Group × Location, or Group × SSD × Location.

For P300 latency, the main effect of Group, main effect of SSD, and all interactions were not significant.

For the N2 amplitudes, there was no significant group difference found in N2 amplitudes [*F*(1,55) = 0.65, *P* = 0.42]. There was a significant main effect of SSD [*F*(2, 110) = 3.56, *P* < 0.05] and N2 amplitudes increased when SSD was longer. Post hoc *t* tests with Bonferroni correction showed that the SSD of 300 ms elicited much larger N2 amplitudes than the SSD of 100 ms (*P* = 0.04). The main effect of Location was also significant [*F*(2,110) = 44.05, *P* < 0.001] as N2 amplitudes had a distribution in frontal sites with a maximum of − 0.89 ± 0.30 µV at Fz. The interaction of SSD × Location was significant [*F*(4,220) = 17.13, *P* < 0.001], whereas interactions of Group × SSD, Group × Location, or Group × SSD × Location were not significant.

**Fig. 3:**
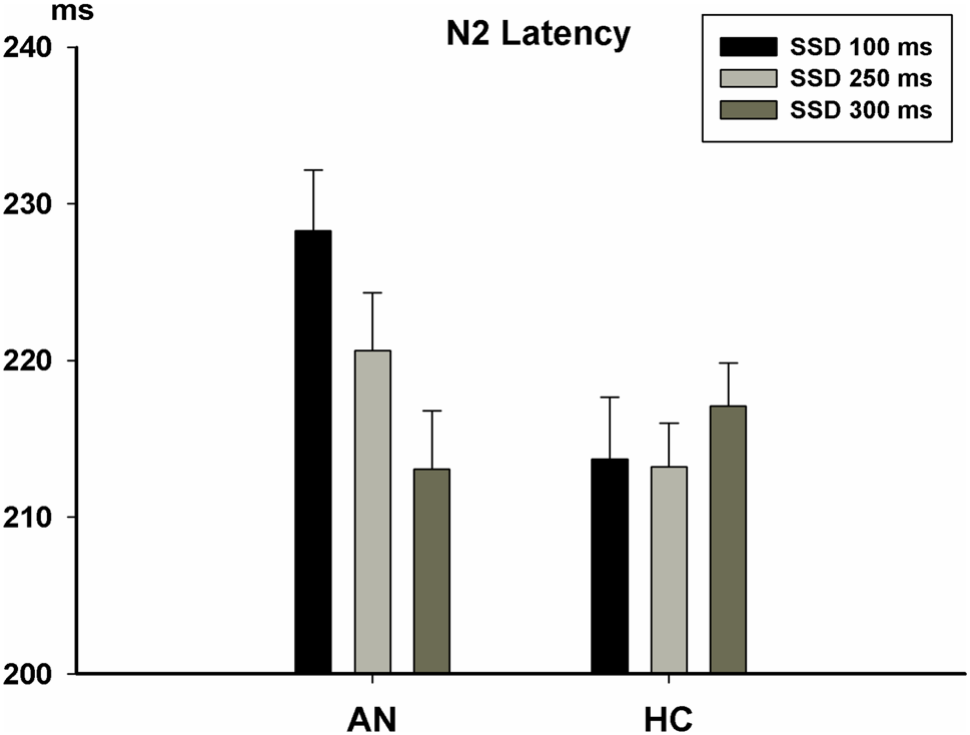
The significant interaction of Group and SSD on N2 latencies

For N2 latencies, we observed significant Group differences [*F*(1,55) = 5.50, *P* < 0.05]. The N2 latencies of the AN group (215.96 ± 2.43 ms) were significantly longer than the HC group (208.09 ± 2.31 ms). The main effect of SSD was not significant [*F*(2, 110) = 0.71, *P* = 0.49]. The main effect of Location was significant [*F*(2,110) = 13.47, *p* < 0.01] with shorter N2 latency at Fz than at FCz (*P* < 0.001) and Cz (*P* = 0.001). There was also a significant interaction of Group and SSD [*F*(2,110) = 3.33, *P* < 0.05]. The N2 latencies in the AN group were sensitive to SSD settings [*F*(2, 52) = 3.32, *P* < 0.05] and shortened with increased SSD (Fig. 3). However, the N2 latencies in HC did not vary according to SSD [*F*(2, 58) = 0.55, *P* = 0.56]. The interactions of Group × Location, SSD × Location, or Group × SSD × Location were not significant.

### Correlations between behavioural and ERP variables

For informational purposes, correlation analyses were conducted between the behavioural accuracy and N2 and P300 amplitudes and latencies at Fz, FCz, and Cz in each SSD condition. Only for SSD of 100 ms, behavioural accuracy was inversely correlated with N2 amplitude (*r* = − 0.64, *P* = 0.002) and P300 amplitude (*r* = − 0.54, *P* = 0.005), and positively correlated with N2 latency (*r* = 0.57, *P* = 0.009) at Cz. Although the majority of these coefficients were not statistically significant, mean correlation coefficients between accuracy and N2 amplitudes (*r* = − 0.347), P300 amplitude (*r* = − 0.305) and N2 latencies (*r* = 0.316) indicated a tendency that higher behavioural accuracy was along with more prominent N2 and P300 amplitudes and longer N2 latencies.

### Correlations between N2/P300 and clinical characteristics

In AN patients, a positive correlation between the N2 amplitudes and the total scores on the EAT-26 was found (Table 3). Patients with AN had enhanced N2 amplitudes with higher scores on the EAT-26. There was also a significant correlation between BMI and N2-latency/P300-amplitude (Table 3). N2 latencies at the 250 ms SSD were negatively correlated with BMI, which means that N2 latencies were prolonged with lower weight AN patients. P300 amplitudes were positively correlated with BMI, indicating AN patients with lower BMI tended to have more attenuated P300 amplitudes. In healthy controls, there were no significant correlations between any N2/P300 components and EAT-26 scores or BMI (Supplementary Table 1). No other relationships were observed.

**Table 3:** Correlation between N2/P300 activities and clinical characteristics in the AN group

	SSD 100 ms	SSD 250 ms	SSD 300 ms
EAT-26 and N2 amplitudes			
Fz	*r* = 0.55, *P* < 0.01**	*r* = 0.47, *P* < 0.05*	*r* = 0.28, *P* = 0.21
FCz	*r* = 0.60, *P* < 0.01**	*r* = 0.49, *P* < 0.05*	*r* = 0.50, *P* < 0.05*
Cz	*r* = 0.54, *P* < 0.05*	*r* = 0.36, *P* = 0.11	*r* = 0.43, *P* < 0.05*
EAT-26 and N2 latencies			
Fz	*r* = − 0.01, *P* = 0.95	*r* = − 0.40, *P* = 0.07	*r* = − 0.06, *P* = 0.80
FCz	*r* = 0.08, *P* = 0.71	*r* = − 0.46, *P* < 0.05*	*r* = 0.16, *P* = 0.49
Cz	*r* = − 0.08, *P* = 0.72	*r* = 0.23, *P* = 0.31	*r* = − 0.09, *P* = 0.68
EAT-26 and P300 amplitudes			
Fz	*r* = 0.33, *P* = 0.12	*r* = 0.23, *P* = 0.29	*r* = 0.28, *P* = 0.20
FCz	*r* = 0.31, *P* = 0.15	*r* = 0.28, *P* = 0.19	*r* = 0.42, *P* < 0.05*
Cz	*r* = 0.27, *P* = 0.21	*r* = 0.23, *P* = 0.29	*r* = 0.15, *P* = 0.50
EAT-26 and P300 latencies			
Fz	*r* = − 0.17, *P* = 0.45	*r* = − 0.21, *P* = 0.33	*r* = 0.02, *P* = 0.91
FCz	*r* = − 0.30, *P* = 0.17	*r* = − 0.38, *P* = 0.07	*r* = − 0.03, *P* = 0.90
Cz	*r* = − 0.26, *P* = 0.24	*r* = − 0.34, *P* = 0.11	*r* = − 0.1, *P* = 0.97
BMI and N2 amplitudes			
Fz	*r* = 0.03, *P* = 0.88	*r* = − 0.03, *P* = 0.90	*r* = 0.16, *P* = 0.43
FCz	*r* = 0.14, *P* = 0.50	*r* = 0.11, *P* = 0.58	*r* = 0.10, *P* = 0.61
Cz	*r* = 0.18, *P* = 0.38	*r* = 0.16, *P* = 0.43	*r* = 0.18, *P* = 0.37
BMI and N2 latencies			
Fz	*r* = − 0.18, *P* = 0.38	*r* = − 0.52, *P* < 0.01**	*r* = − 0.23, *P* = 0.25
FCz	*r* = − 0.20, *P* = 0.33	*r* = − 0.38, *P* < 0.05*	*r* = − 0.01 *P* = 0.95
Cz	*r* = − 0.36, *P* = 0.07	*r* = − 0.38, *P* < 0.05*	*r* = − 0.34, *P* = 0.08
BMI and P300 amplitudes			
Fz	*r* = 0.12, *P* = 0.56	*r* = 0.25, *P* = 0.06	*r* = 0.29, *P* < 0.05*
FCz	*r* = 0.17, *P* = 0.40	*r* = 0.27, *P* < 0.05*	*r* = 0.45, *P* < 0.01**
Cz	*r* = 0.22, *P* = 0.27	*r* = 0.23, *P* = 0.08	*r* = 0.25, *P* = 0.06
BMI and P300 latencies			
Fz	*r* = 0.11, *P* = 0.59	*r* = 0.15, *P* = 0.47	*r* = 0.28, *P* = 0.17
FCz	*r* = 0.26, *P* = 0.19	*r* = − 0.03, *P* = 0.89	*r* = 0.40, *P* < 0.05*
Cz	*r* = − 0.13, *P* = 0.52	*r* = 0.22, *P* = 0.27	*r* = 0.49, *P* < 0.01**

Uncorrected *P* values are presented in the table

*P < 0.05, **P < 0.01

## Discussion

We examined response inhibition in unmedicated AN patients using comparisons to HCs with the stop-signal tasks. To the best of our knowledge, our study is the first study to explore electrophysiological activity during response inhibition in AN patients across different levels of inhibitory demands. Partially validating our first hypothesis, our results showed that the Stop signal evoked lower P300 amplitudes and longer N2 latencies in AN as compared to HCs, suggesting deficits in inhibitory control in AN. However, contrary to the second hypothesis, we found that N2 latencies were reduced in AN group when the inhibitory demand was increased, whereas there were no differences in N2 latencies across three SSD durations in HCs. In addition, there were no significant interactions between group and inhibitory demand for N2 amplitude, P300 amplitude, or P300 latency. Such observation indicate that the processing of the Stop signal may be faster for difficult loads than for easy loads in AN. In other words, individuals with AN may improve their inhibition function in the face of difficult condition, supporting a more complex inhibitory control processes [10]. All of these results showed significant differences in neural activity between the AN and the HC groups during response inhibition.

Our performance findings showed that no group difference existed in Stop trials. The lack of a between-group difference in inhibitory behavioural measures was consistent with previous studies [6, 16]. In contrast, another study reported poor inhibitory performance on the stop-signal task in AN compared to HC individuals [13]. The discrepancy may be due to the task design. The previous study design resulted in uniform difficulty adjusted for individual performance. However, in the current study, the stop stimulus presentation time was designed on set a pattern of varied inhibitory difficulty across trials. Furthermore, although the accuracy measure was generally not significantly correlated, we still find some correlation between accuracy and N2/P300 amplitudes which need to be explored in more samples.

Studies in HCs found that the stop-signal-evoked P300 was associated with motor inhibition and reflected the monitoring of the outcome of the inhibitory process [30]. Attenuated P300 amplitudes might reflect impaired response inhibition in patients with AN. There was also a main effect of SSD duration on the P300 amplitudes. The P300 amplitudes with an SSD of 300 ms were lower compared to those with the SSDs of 100 to 250 ms. These results were consistent with previous studies on the stop-signal-evoked P300 [26]. The accuracy for the SSDs of 300 ms was lower compared to the SSDs of 100 to 250 ms. Larger P300 activities were observed with successful response inhibitions than failed ones [29]. Although no stop-signal-evoked P300 activation has been reported in AN individuals yet, studies in HCs found that large P300 amplitudes elicited by the Stop signal reflect greater effective inhibition [26, 29]. Due to the deficits in cognitive control, AN patients might have difficulties correctly evaluating the output of stopping (i.e., giving up food intake without thinking about the outcomes for physical health). In addition, we found attenuated P300 amplitudes with lower BMIs in AN patients. The correlations should be interpreted with caution, because amplitude not only reflects the neural activity but are also related to BMI [37]. Future studies may explore the correlation between the ERP component and BMI in recovered patients. Besides, we did not find group differences on P300 latency, it may reflect the temporal properties of process during P300 was less affected.

In our study, we found that N2 amplitudes increased with longer SSD in all subjects. Furthermore, we observed a significant interaction of Group and SSD on N2 latencies. Stop-signal-evoked N2 was associated with an evaluative process that detected the occurrence of conflict between the go and inhibit responses [26]. In normal adults, larger N2 amplitudes were considered to be associated with enhanced inhibitory processing [29]. Recently, a study in healthy adults reported a larger N2 amplitude when inhibiting responses elicited by high-calorie stimuli, indicating that high-calorie foods required increased recruitment of inhibitory control processes [38]. Linked to our study, to complete a large SSD task that represented higher-order processing, participants needed a greater “degree” of inhibitory process activation, manifesting in larger N2 amplitudes. Although no group difference on N2 amplitude was found in our study, it may indicate that the impaired inhibitory neural activity in AN patients may be affected more on temporal properties to reach the equal “degree” of neural activation during N2 compared to HCs. Additionally, and more remarkably, patients with AN had delayed N2 latencies compared to HCs, reflecting their impaired response inhibition with the consumption of more attentional resources. In particular, shorter N2 latencies in AN patients found for difficult inhibit trials were comparable to HC N2 latencies for easy inhibit trials. The alteration in N2 latency along with demand load can be interpreted by greater task efficiency when the demand is high, indicating faster processing. This finding is in line with clinical observations that AN patients have an elevated ability to resist temptations not only of food but also of most comforts and pleasures in life [12, 39]. A previous fMRI study using a similar task found a less mPFC activity in the high demand task, indicating less activation needed to complete the task compared with HCs, and was also interpreted as higher efficiency [6]. The altered frontal N2 activity with high inhibitory load might contribute to accentuated inhibition and control in AN patients [6]. Our finding of significant interaction between group and task demand on N2 latency rather than N2/P300 amplitude or P300 latency suggested that the impaired inhibitory processing in AN may partly affect the N2 latency, but not all the parts of the ERP components. Our study suggested an aberrant neural activation underlying elevated cognitive control in AN, which was in line with over-control of food intake in AN [40].

Our findings suggested that both superior and inferior inhibitory control process could be present in individuals with AN. On the one hand, shorter P3 amplitude and prolonged N2 latency in the AN group may be suggestive of poor inhibitory control, and on the other hand, the interaction between SSD and group on N2 latency may be suggestive of increased inhibitory control on difficult trials. Previous studies also found similar conflicting findings [10, 41]. For example, one study reported that AN patients have both impulsivity in behavioural measures and over-control in neuropsychological tests [10]. Indeed, some behavioural studies found evidence for lack of control [13, 42], whereas others reported lower impulsivity and increased inhibitory control [9, 11, 12]. Hence, our data supported the view that there was a complex underlying mechanism contributing to the disease [10]. Furthermore, our data suggested that the deficit in inhibition cancellation in AN may be attributed to the different context. These findings supported the idea that altered self-control in the disorder might be limited to demand-specific tasks [6].

Source analyses suggested that the fronto-central N2 activity was generated in the prefrontal area [26, 30], which is included in the neural network associated with cognitive control [43]. Altered activation over prefrontal regions has been found in AN patients by functional MRI studies [6, 17, 44]. Top-down hyperactivity over the prefrontal cortex in conjunction with reduced bottom-up hypoactivity within the limbic neurocircuit, including the striatum, the hippocampus and the amygdala, was observed in females with AN when viewing rewarding stimuli [1, 7, 17, 20]. However, reduced prefrontal activity was also revealed in AN patients [6, 7]. Activation in the medial prefrontal cortex was reduced during trials when the inhibitory demand was high, whereas it was comparable when inhibitory demand was low, reflecting a demand-specific alteration in recovered anorexic women [6]. Altered function of the prefrontal cortex was modulated by inhibitory demand levels, which may play an important role in the over-control of inhibition in AN patients but needs more evidence.

N2 activity seems sensitive to clinical characteristics in AN patients. We found a positive correlation between N2 amplitudes and scores on the EAT-26, indicating that AN individuals with severe symptoms (high EAT-26 score) may have stronger neural activity related to inhibition. Our results were consistent with the other ERP study in AN patients, which found a positive correlation between body dissatisfaction scores and P3a amplitudes [33]. Recent studies in healthy people found larger N2 amplitudes were present when inhibiting high-calorie foods but not low-calorie foods [38], and N2 amplitudes predicted increased food intake [32]. Our results supported enhanced inhibitory processing in AN which was related to the severity of the disease. In addition, a negative correlation between N2 latencies and BMI was found, although only in 250 ms SSD trails (medium difficulty), indicating under normal condition, lower BMI with AN may need more time to process inhibition. Although most studies have reported only alterations in amplitude, our delayed N2 latencies in AN might be the temporal indication of the deficits in initiating response inhibition. Such interpretation should be investigated further by clarifying the complexity role of N2 component in different task setting and BMI. In summary, previous studies in both eating disorder and healthy adults showed alterations in N2 components related to inhibiting food and shape stimuli, and severe AN patients in our study tended to have attenuated N2 activity, suggesting deficits in response inhibition.

There are some limitations in the present study. First, the sample size of the AN group was small. Second, the Go (a circle) and Stop (a cross) signal in our study are general stimulus. Future studies using paradigms with disorder-specific stimuli may help to clarify the role of inhibition control in AN, such as high-calorie foods vs low-calorie foods, or thin body image vs fat body image. Third, we only recruited female in the study whereas AN prevalence in men is also common, male patients with AN should be included in the future to explore the sex differences in inhibition control in AN.

## Conclusions

In the present study, we demonstrated preliminary evidence of aberrant neural processing related to inhibition control in currently ill AN patients. Altered P300 amplitudes and N2 latencies supported impaired neural processing of response inhibition in AN. In particular, alterations in fronto-central N2 latencies were demand-related, suggesting a complicated inhibitory control processes in AN. Based on findings from our study and the neuroimaging literature [6, 23], the prefrontal cortex has been suggested to have a crucial role in AN. New non-invasive neurostimulation treatment, such as transcranial direct current stimulation (tDCS) and transcranial magnetic stimulation (TMS), specifically targeted to this region may improve outcomes.

## Electronic supplementary material

Below is the link to the electronic supplementary material.

Supplementary material 1 (DOCX 14 KB)
