# Supplementary material for: Deficits in response inhibition on varied levels of demand load in anorexia nervosa: an event-related potentials study
Source: Eat Weight Disord. 2018 Aug 28;25(1):231–40. doi: 10.1007/s40519-018-0558-2 (PMC6997249; doi:10.1007/s40519-018-0558-2)
Supplement: Supplementary file 1 — Supplementary material 1 (DOCX 14 KB) [file 40519_2018_558_MOESM1_ESM.docx]

Supplementary Table 1. Correlation between N2/P300 activity and clinical characteristics in the HC group

|  | SSD 100 ms | SSD 250 ms | SSD 300 ms |
| --- | --- | --- | --- |
| *EAT-26 and N2 amplitudes* | | | |
| Fz | r=0.20, P=0.28 | r=-0.12, P=0.53 | r=-0.11, P=0.58 |
| FCz | r=-0.03, P=0.86 | r=-0.24, P=0.20 | r=-0.15, P=0.44 |
| Cz | r=-0.14, P=0.47 | r=-0.27, P=0.15 | r=-0.11, P=0.56 |
| *EAT-26 and N2 latencies* | | | |
| Fz | r=0.01, P=0.95 | r=-0.11, P=0.57 | r=-0.23, P=0.21 |
| FCz | r=-0.06, P=0.76 | r=-0.07, P=0.73 | r=-0.01, P=0.95 |
| Cz | r=0.01, P=0.98 | r=0.02, P=0.93 | r=0.03, P=0.87 |
| *EAT-26 and P300 amplitudes* | | | |
| Fz | r= 0.21, P=0.91 | r=0.16, P=0.93 | r=0.15, P=0.42 |
| FCz | r=0.13, P=0.50 | r=0.24, P=0.90 | r=0.04, P=0.84 |
| Cz | r=0.18, P=0.35 | r=0.10, P=0.60 | r=0.03, P=0.87 |
| *EAT-26 and P300 latencies* | | | |
| Fz | r= 0.18, P=0.33 | r=-0.32, P=0.09 | r=-0.19, P=0.33 |
| FCz | r=0.20, P=0.30 | r=0.01, P=0.98 | r=0.08, P=0.68 |
| Cz | r=0.12, P=0.54 | r=0.10, P=0.61 | r=0.31, P=0.10 |
| *BMI and N2 amplitudes* | | | |
| Fz | r=-0.01, P=0.95 | r=-0.02, P=0.93 | r=-0.05, P=0.78 |
| FCz | r=-0.15, P=0.44 | r=-0.09, P=0.62 | r=-0.01, P=0.97 |
| Cz | r=-0.08, P=0.66 | r=-0.08, P=0.67 | r=0.00, P=0.99 |
| *BMI and N2 latencies* | | | |
| Fz | r=0.17, P=0.37 | r=-0.11, P=0.58 | r=-0.06, P=0.75 |
| FCz | r=0.14, P=0.47 | r=-0.09, P=0.64 | r=-0.00 P=0.98 |
| Cz | r=0.04, P=0.84 | r=0.03, P=0.88 | r=-0.05, P=0.81 |
| *BMI and P300 amplitudes* | | | |
| Fz | r=0.10, P=0.62 | r=0.06, P=0.74 | r=0.22, P=0.91 |
| FCz | r=-0.00, P=0.99 | r=-0.07, P=0.73 | r=0.11, P=0.56 |
| Cz | r=-0.05, P=0.78 | r=-0.20, P=0.28 | r=-0.10, P=0.61 |
| *BMI and P300 latencies* | | | |
| Fz | r= 0.21, P=0.26 | r=-0.20, P=0.30 | r=-0.29, P=0.12 |
| FCz | r=0.14, P=0.47 | r=-0.24, P=0.21 | r=-0.03, P=0.88 |
| Cz | r=-0.10, P=0.61 | r=-0.25, P=0.18 | r=0.09, P=0.64 |
